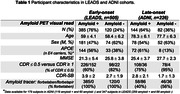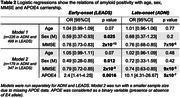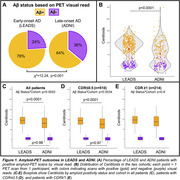# Amyloid‐PET in patients with a clinical diagnosis of sporadic early‐ versus late‐onset AD: comparison of the LEADS and ADNI cohorts

**DOI:** 10.1002/alz.094101

**Published:** 2025-01-09

**Authors:** Julien Lagarde, Piyush Maiti, Daniel R. Schonhaut, Jiaxiuxiu Zhang, David N. soleimani‐meigooni, Ehud Zeltzer, Charles Windon, Maison Abu Raya, Agathe Vrillon, Dustin B. Hammers, Jeffrey L. Dage, Kelly N. Nudelman, Ani Eloyan, Robert A. Koeppe, Susan M. Landau, Maria C. Carrillo, Alexandra Touroutoglou, Prashanthi Vemuri, Bradford C. Dickerson, Liana G. Apostolova, Gil D. Rabinovici, Renaud La Joie

**Affiliations:** ^1^ Department of Neurology, University of California, San Francisco, San Francisco, CA USA; ^2^ Memory and Aging Center, Weill Institute for Neurosciences, University of California, San Francisco, San Francisco, CA USA; ^3^ University of california San Francisco, San Francisco, CA USA; ^4^ Memory and Aging center, UCSF, San Francisco, CA USA; ^5^ Indiana University School of Medicine, Indianapolis, IN USA; ^6^ Department of Neurology, Indiana School of Medicine, Indianapolis, IN USA; ^7^ Department of Neurology, Indiana University School of Medicine, Indianapolis, IN USA; ^8^ Department of Biostatistics, Brown University, Providence, RI USA; ^9^ University of Michigan, Ann Arbor, MI USA; ^10^ University of California, Berkeley, Berkeley, CA USA; ^11^ Alzheimer’s Association, Chicago, IL USA; ^12^ Department of Neurology, Harvard Medical School, Boston, MA USA; ^13^ Mayo Clinic, Rochester, MN USA; ^14^ Department of Neurology, Memory and Aging Center, University of California San Francisco, San Francisco, CA USA

## Abstract

**Background:**

Large‐scale studies comparing sporadic early‐onset AD (EOAD, age<65) and late‐onset AD (LOAD, age = 65) are lacking. We compared amyloid‐PET outcomes (positivity rate and amyloid burden) between patients clinically diagnosed with sporadic EOAD vs LOAD, leveraging data from the Longitudinal Early‐Onset AD Study (LEADS) and the Alzheimer’s Disease Neuroimaging Initiative 3 (ADNI3).

**Method:**

731 patients meeting the 2011 NIA‐AA criteria for AD dementia or MCI were included (505 early‐onset from LEADS, 226 late‐onset from ADNI3, Table 1). All participants underwent amyloid‐PET with [18F]Florbetaben or [18F]Florbetapir. Amyloid positivity was centrally determined by a process involving a visual read by a trained expert and PET‐only quantification; in case of a discrepancy, a read from an independent physician acted as a tiebreaker. Logistic regressions in each cohort examined relations between amyloid positivity and age, sex, MMSE and APOE4 genotype. Amyloid burden was independently quantified in Centiloids using an MRI‐based pipeline. Mean Centiloids in LEADS and ADNI were compared with two‐way ANOVA, for visually positive and visually negative scans.

**Result:**

Amyloid positivity rate was higher in LEADS (76%) than ADNI (64%, p<0.001, Figure 1A). Lower MMSE and APOE4 genotype increased odds of amyloid positivity in both cohorts, although the APOE4 effect was stronger in ADNI than LEADS (OR = 10.1 versus 2.4, p = 0.007, Table 2). Amyloid positivity was more common in females across cohorts, but this effect was only statistically significant in LEADS (Table 2). Centiloids were bimodally distributed in both cohorts, although the separation between positive and negative scans was more prominent in LEADS (Figure 1B). Visually positive scans had significantly higher Centiloids in LEADS than in ADNI, whereas no cohort difference was observed for visually negative scans (Figure 1C). Sensitivity analyses showed that this effect was driven by patients with MCI (CDR = 0.5; Figure 1D‐E).

**Conclusion:**

The lower amyloid positivity rate in ADNI might be due to AD‐mimicking pathologies being more common at an older age. The higher amyloid burden in early‐onset, amyloid‐positive patients could reflect younger patients being diagnosed later in the disease course compared to typical, late‐onset patients. Alternatively, younger patients might tolerate higher neuropathology burden due to higher brain reserve or fewer co‐pathologies.